# Longitudinal Association between Maternal Autonomy Support and Controlling Parenting and Adolescents’ Depressive Symptoms

**DOI:** 10.1007/s10964-022-01722-1

**Published:** 2023-01-19

**Authors:** Ayumi Tanaka, Ayame Tamura, Ryo Ishii, Shin-ichi Ishikawa, Naoki Nakazato, Kazuhiro Ohtani, Michiko Sakaki, Takashi Suzuki, Kou Murayama

**Affiliations:** 1 Doshisha University, Kyotanabe, Japan; 2 University of Human Environments, Okazaki, Japan; 3 Nara University of Education, Nara, Japan; 4 Oita University, Oita, Japan; 5 Hokkaido University, Sapporo, Japan; 6 Kouchi University of Technology, Kami, Japan; 7 University of Tübingen, Tübingen, Germany

**Keywords:** Adolescents, Autonomy support, Controlling parenting, Basic psychological needs satisfaction, Depressive symptoms, Self-determination theory

## Abstract

Most studies on autonomy support and controlling parenting rely on children’s perceptions, despite the limitations of this approach. This study investigated congruency between autonomy support and controlling parenting reported by mothers and adolescents and their association with adolescents’ depressive symptoms via basic psychological needs satisfaction. Participants included 408 Japanese mother–adolescent (*M*_age_ = 13.73, *SD* = 0.90, 52% female) pairs who completed a questionnaire at two time points four months apart. Results demonstrated low to moderate levels of mother–adolescent agreement. Cross-lagged regression models revealed that mothers’ reported autonomy support positively predicted adolescents’ basic psychological needs satisfactions, which was negatively associated with depressive symptoms. The independent roles of parenting reported by mothers and adolescents for adolescents’ well-being were discussed.

## Introduction

Researchers have traditionally emphasized the importance of family in adolescents’ dynamic developmental process. Since Grolnick and Ryan’s conceptualization and operationalization of autonomy support within the self-determination theory framework (e.g., Grolnick & Ryan, 1989; Ryan & Deci, 2017), research has underscored the importance of parental autonomy support on adolescents’ growth and healthy functioning. However, most studies have relied exclusively on children’s perception of parenting (Cheung et al., 2016). The present study aimed to shed light on incongruence in mothers’ report and adolescents’ perception of parenting in the context of self-determination theory and to examine their possible independent roles on adolescents’ basic psychological needs satisfaction, the condition assumed to be essential to adolescents’ optimal development and to mediate the link between parental behavior and adolescents’ well-being outcomes.

### Autonomy Support and Controlling Parenting: Self-determination Perspective

Self-determination theory identifies autonomy support and controlling behavior as being two of the most important dimensions of parenting. Autonomy support refers to behaviors that support ones’ experience of autonomy, including providing choices, perspective taking, careful listening, and providing of rationales for engaging in a particular behavior (Deci & Ryan, 1987; Pomerantz et al., 2007). Controlling parenting refers to behaviors that induce or pressure children to do things they would not freely do (Deci & Ryan, 1987), including valuing obedience and conformity first, controlling use of rewards, and imposing the parents’ own agenda on the child or allowing few choices (Grolnick & Pomerantz, 2009; Grolnick & Ryan, 1989). The two behaviors are not on one continuum (Cheung et al., 2016); it is possible to be high in one but not necessarily low in the other. Some parents may do both, while others may not engage in either behavior much.

### Parenting Behavior, Adolescents’ Basic Psychological Needs Satisfaction, and Depressive Symptoms

Parental autonomy support and controlling behavior have been found to be negatively and positively associated with risk for mental illness in adolescence, respectively (see Vasquez et al., 2016), including depressive symptoms. Adolescents’ perceived parental autonomy support was negatively related with depressive symptoms across three major educational transitions: middle school, high school, and post high school (Duineveld et al., 2017). Maternal controlling parenting predicted adolescents’ depressive symptoms consistently from 13 to 17 years of age (Werner et al., 2016). Longitudinal negative associations were found between perceived parental autonomy support and early and middle adolescents’ depressive symptoms, while perceived autonomy support from best friends was not significantly associated with depressive symptoms (Van der Giessen et al., 2014). These results highlight the particular importance of parental autonomy support during adolescence.

Self-determination theory proposes that parental autonomy support and controlling behaviors positively and negatively influence their children’s satisfaction of basic psychological needs, respectively. Satisfaction in feeling autonomy, competence, and relatedness are “nutrients that are essential for growth, integrity, and well-being” (Ryan & Deci, 2017, p. 10), and relate to motivational, affective, cognitive, and other psychological full-functioning. Autonomy refers to the need to self-endorse one’s actions. Competence refers to the need to feel effectance, mastery, and able to operate effectively within one’s important life contexts (see also White, 1959). Relatedness refers to the need to feel socially connected. Self-determination theory assumes that satisfaction of basic psychological needs increases adolescents’ vitality and life satisfaction and enhances mental wellness, and lack of satisfaction leads to lowered vitality, loss of volition, greater fragmentation, and ill-being. The satisfaction of these basic psychological needs is facilitated or undermined critically in autonomy-supportive or -thwarting environments (Ryan & Deci, 2017). Since family influences adolescents the most significantly (Wigfield et al., 2011), the effect of parental behavior can be well-understood by considering the mediation of adolescents’ satisfaction of basic psychological needs. For example, when parents support their adolescents’ autonomy, such as allowing freedom to choose their own activities, adolescents feel satisfaction of the need for autonomy, such as feeling free to express their own ideas; need for competence, such as feeling a sense of accomplishment with what they do; and need for relatedness, such as feeling that people in their life care about them (Ryan & Deci, 2019), which enhances their well-being. Conversely, when parents show controlling behavior, such as threating punishment for disobedience, adolescents feel less satisfaction of the need for autonomy, such as having lowered feelings of ownership in their actions; need for competence, such as feeling they are not capable; and need for relatedness, such as feeling they do not like the people they interact with, which leads to mental ill-being.

Self-determination theory assumes the processes are universal, and numerous studies of adolescents support the assumption in a wide range of cultural contexts (Ryan & Deci, 2017). For example, satisfaction of basic psychological needs contributed to vitality and life satisfaction of adolescents in Belgium, China, the USA, and Peru, regardless of their cultural background (Chen et al., 2015). In research on Italian adolescents, perceived parental autonomy support and control were associated with vitality and depression, respectively, and the relation was mediated by satisfaction and frustration of basic psychological needs, respectively (Costa et al., 2016). A recent meta-analysis showed that no differences in autonomy’s importance to well-being between East Asian and North American samples (Yu et al., 2018).

### Parenting Behavior Reported by Adolescents vs. Parents

Studies on parental autonomy support and control have predominantly relied on children’s perceptions of parenting (see Pinquart, 2017; Vasquez et al., 2016; Yap et al., 2014, for meta-analyses). Parenting literature, including studies within the self-determination theory framework, has traditionally assumed that children are more influenced by their perception and interpretation of parental behaviors than actual parental behaviors or those reported by the parents (e.g., Deci & Ryan, 1987; Demo et al., 1987; Pinquart, 2017; Schaefer, 1965). However, there are two important issues to consider with this approach.

First, those studies paid relatively little attention to the fact that parents and children have overlapping but dissociated perceptions of parenting behaviors (De Los Reyes & MacCauley, 2016; Taber, 2010). For example, a meta-analysis including 80 studies of mother–child dyads demonstrated that the parent–child correlation across the parenting constructs ranges from 0.23 to 0.28 of the Pearson *r* effect sizes (Korelitz & Garber, 2016). To the best of our knowledge, there is only one study explicitly shedding light on agreement in parental autonomy support and controlling behavior in the context of self-determination theory. In a study of mothers and their adolescents, Cheung et al. (2016) reported low to modest correlations between mothers’ and adolescents’ reports in China (*r* = 0.23 for autonomy support and 0.28 for control) and the United States (*r* = 0.17 for autonomy support and 0.48 for control). No difference in correlations across countries was reported, implying the universality of the low agreement phenomenon. However, there is a clear need for more research in this area.

Second, there is little, if any, multi-informant research examining the relative impact of autonomy support and controlling parenting on adolescents’ basic psychological needs satisfaction, which is expected to mediate the link between parental behavior and adolescents’ psychological outcomes. Scarce multi-informant studies in self-determination theory have examined only the direct relationship between parenting and adolescent outcomes, and the findings have been mixed regarding whether parents’ or adolescents’ report contributes more to those outcomes (Nelemans et al., 2020; Vrolijk et al., 2020). For example, in research on parenting behavior rated by adolescents, mothers, and fathers (Janssens et al., 2015), parental psychological control was shown to be related to adolescents internalizing and externalizing problem behaviors, regardless of the respondent. Moreover, adolescents’ depressive symptoms were predicted by parent-reported, not adolescents’ perceived, parental support. In research on American and Chinese adolescents (Cheung et al., 2016), regardless of the country, adolescents’ reports of maternal autonomy support and controlling parenting were positively and negatively associated with their emotional functioning, respectively. No association between mothers’ reported autonomy support and emotional functioning was found. These studies might overlook the possibility that parents’ reported and adolescents’ perceived parenting are differently related with adolescents’ basic psychological needs satisfaction and cause mixed findings. It is necessary to understand the relative impact of parents’ reported and adolescents’ perceived autonomy support and controlling parenting on basic psychological needs satisfaction, as well as how it mediates the association between parents’ reported and adolescents’ perceived parenting behavior and outcomes. This understanding would further clarify the importance of parenting in the adolescent developmental process and adolescent perception thereof.

## Current Study

Reviewed studies showed that high parental autonomy support and low controlling parenting contribute to decreased risk for adolescent depressive symptoms through enhancing satisfaction of basic psychological needs. However, most studies relied on children’s perceptions, ignoring findings from multi-informant studies that parents’ and children’s perception of parenting behaviors might not be redundant but unique. Moreover, there is a lack of research examining the relative impact of autonomy support and controlling parenting reported by parents and adolescents on adolescents’ basic psychological needs satisfaction, which is necessary to elucidate the process of the influence of parenting behaviors.

The present study’s first goal was to examine agreement between adolescents’ perceptions of parenting behaviors and the parenting behaviors reported by parents^1^. Moderate correlations were expected (Hypotheses 1), based on previous multi-informant studies. The second goal was to investigate whether parents’ and adolescents’ perspectives are differentially related to basic psychological needs satisfaction. Following the traditional assumption mentioned earlier, this research hypothesized a stronger association of adolescents’ perceived parenting compared to parents’ reported parenting. Specifically, autonomy support and controlling parenting perceived by adolescents would positively and negatively predict basic psychological needs satisfaction, respectively (Hypotheses 2). This study also aimed to replicate the well-supported findings that a higher level of adolescents’ basic psychological needs satisfaction would lead to a lower level of depressive symptoms (Hypotheses 3).

## Methods

### Participants

This study was conducted as a part of a larger two-wave longitudinal research project for junior high school students (7th to 9th graders) and their mothers living in Japan. It focused on maternal parenting, given mothers’ strong central role in caregiving and socializing adolescents in Japan (Kayama, 2010). Participants were recruited through a private research firm, the Japan Management Association. The firm exchanged contracts with the participants regarding consent to participate in studies and monetary compensation when participants registered with the database. Upon the firm’s announcement and explanation of the current study, only those who agreed to participate responded to the recruitment. A total of 408 mothers and their children (213 girls) agreed to participate in the first wave of data collection (T1). The adolescents’ average age at T1 was 13.73 years (*SD* = 0.90), and that of the mothers was 44.84 years (*SD* = 4.49). The annual family income was distributed from low (< JPY 2 million, 3.5%) to high (>12 million, 1.2%), and the median was the point between JPY 6 million and 6.99 million (see Appendix, Table 4). In the second wave of data collection (T2), a total of 373 mother–child pairs (194 girls) remained to complete the study. The dataset used in this study is also used in study by Kurdi et al. (2022) to investigate basic need satisfaction in parents and adolescents.

### Procedure

Participants completed the same set of questionnaires twice. They received their first questionnaire package in October 2019 (T1) and the second package in February–March 2020 (T2). Data collection was completed before the end of the school year and before the significant impact of COVID-19 in Japan. The relatively short four-month period was appropriate for the present study as it focuses on change of depressive symptoms and some researchers report that stability of depressive symptoms is not high from childhood through adolescence (e.g., Pihlakoski et al., 2006).

Packages were sent by regular mail and participants were asked to return them after completion. At both T1 and T2, the mothers and adolescents were instructed to complete their questionnaire independently, not look at each other’s answers, and put them in separate envelopes when returning them. The study was approved by the “Research on Humans” Ethical Committee of the first author’s university (number 19041).

### Measures

#### Adolescents’ perceptions of mother’s autonomy support and controlling parenting

The present study used the 24-item Perceived Parental Autonomy Support Scale (P-PASS) developed and validated by Mageau et al. (2015). All the items pertain to directly observable behaviors. It includes 12 items on autonomy support covering three aspects: choice within certain limits (e.g., “My point of view was very important to my parents when they made important decisions concerning me”), rationale for demands and limits (e.g., “My parents made sure that I understood why they forbid certain things”), and acknowledgment of feelings (e.g., “My parents were able to put themselves in my shoes and understand my feelings”). The other 12 items are on controlling parental behavior covering three aspects: threats to punish (e.g., “I always had to do what my parents wanted to do; if not, they would threaten to take away privileges”), performance pressures (e.g., “My parents insisted that I always be better than others”), and guilt-inducing criticisms (e.g., “My parents made me feel guilty for anything and everything”). The items for controlling parenting encompass the traditional concept of psychological control (e.g., Barber, 1994; Schaefer, 1965), while allowing for the possibility that parents are also controlling regarding adolescents’ behaviors (Mageau et al., 2015). The measure was translated from English to Japanese; back translation was conducted and checked by multiple experts. Responses were rated on a seven-point Likert scale, ranging from 1 = *do not agree at all* to 7 = *very strongly agree*.

Since there was no Japanese study documenting the scale’s psychometric properties, the factor structure was evaluated by applying the same method used by Mageau et al. (2015) (exploratory factor analysis with maximum likelihood and oblimin rotation), using T1 data. Based on a visual scree plot, two factor solutions explaining 50% of the variance were adopted. The first factor accounted for the autonomy support items, and the second factor accounted for the controlling parenting items; each item loaded on its factor with a loading above 0.39. All-cross-loadings were well below the recommended threshold of 0.40 (Stevens, 2002). The full results of the factor analyses are presented in Appendix Table 5.

Autonomy support and controlling parenting scores were obtained by averaging the scores of the items of each subscale, separately for T1 and T2. The Cronbach’s α for autonomy support at T1 and T2 was 0.92 and 0.94, respectively, and for controlling parenting was 0.91 and 0.93, respectively.

#### Mothers’ self-report of autonomy support and controlling parenting

A slightly modified version of the 24-item P-PASS was used to obtain mothers’ self-reports on their own behaviors toward the child (e.g., “My child’s point of view was very important to me when I made important decisions concerning him/her”, “My child always had to do what I wanted to do; if not, I would threaten to take away privileges”). The factor structure of the scale was evaluated applying the same method as with the adolescents’ version. Based on a scree plot, two factor solutions that explained 43% of the variance were adopted. The first factor accounted for the controlling parenting items, and the second factor accounted for the autonomy support items. Each item loaded on its factor with a loading above 0.43, except one loading, 0.28, for an autonomy support item (“I hoped that my child would make choices that corresponded to his/her interests and preferences regardless of what mine were”). All cross-loadings were below the threshold of 0.40. The full results of the factor analyses are presented in Appendix Table 6. Autonomy support and controlling parenting scores were obtained by averaging the item scores of each subscale, separately for T1 and T2. Cronbach’s α for autonomy support at T1 and T2 was 0.87 and 0.88, respectively, and for controlling parenting at T1 and T2, it was 0.91 and 0.93, respectively.

#### Basic psychological needs satisfaction

Adolescents reported their level of basic psychological needs satisfaction by filling out the satisfaction subscale from the Japanese version of the Basic Psychological Need Satisfaction and Frustration Scale (Nishimura & Suzuki, 2016) at T1 and T2. The scale contains four items for the satisfaction of each basic psychological need: autonomy, competence, and relatedness. Responses are rated on a five-point Likert scale ranging from 1 = *completely disagree* to 5 = *completely agree*. The scale has been demonstrated to adequately assess basic psychological needs satisfaction in a Japanese sample (e.g., Nishimura et al., 2021; Xiao & Toyama, 2020). Considering that high correlations between the three subscales have been reported often (e.g., Chen et al., 2015; Campbell et al., 2014) and that the effect of basic psychological needs satisfaction as a whole is the main interest of the present study, composite scores were calculated by averaging scores for all 12 items, separately for each T1 and T2. Cronbach’s α at T1 and T2 was 0.91 and 0.92, respectively.

#### Depressive symptoms

Adolescents reported their level of depressive symptoms using the short version of the Depression Self-Rating Scale for Children (Namikawa et al., 2011) at T1 and T2. The scale comprises two subscales: Decreased Enjoyment and Activities (five items) and Depressive Mood (four items). Many clinical and developmental studies in Japan demonstrated the scale’s high reliability and validity (e.g., Deno et al., 2021; Murayama et al., 2020). Responses are rated on a three-point Likert scale, ranging from 1 = *not at all* to 3 = *always*, and are averaged across all items to form a scale score. Cronbach’s α at T1 and T2 was 0.79 and 0.80, respectively.

#### Mothers’ and adolescents’ reported parent–child relationship satisfaction

Mothers and adolescents each answered two items (“I get along well with my mother/child”; “I am satisfied with the relationship with my mother/child”) from Lin et al. (2013) that measured the extent to which they were satisfied with their relationship with each other. The measure was translated from English to Japanese, and back translation was conducted and checked by multiple experts. A five-point Likert scale, ranging from 1 = *strongly disagree* to 5 = *strongly agree*, was used. The responses for the two items were averaged to form parent–child relationship satisfaction scores from the mothers and adolescents. Cronbach’s α at T1 and T2 was 0.88 and 0.88, for mothers and 0.89 and 0.89 for adolescents, respectively.

Originally, parent–child relationship satisfaction was intended to be a control variable; however, it was removed to avoid complexity in the interpretation of the results^2^. The results were essentially the same regardless of its inclusion. Instead, this measure was used in the imputation model of missing values to add more information and increase the predictive power in imputation, given the correlation between autonomy support and controlling parenting (0.42 and −0.29 in mothers’ report and 0.66 and −0.44 in adolescents’ report at T1).

#### Family socioeconomic status

Each family’s socioeconomic status was evaluated using the annual income reported by the mother. Participants were asked to choose the range of their annual income from 12 options, from 1 = *less than JPY 2 million* to 12 = *more than 15 million* (see Appendix B, Table B).

### Statistical Analyses

**Fig. 1:**
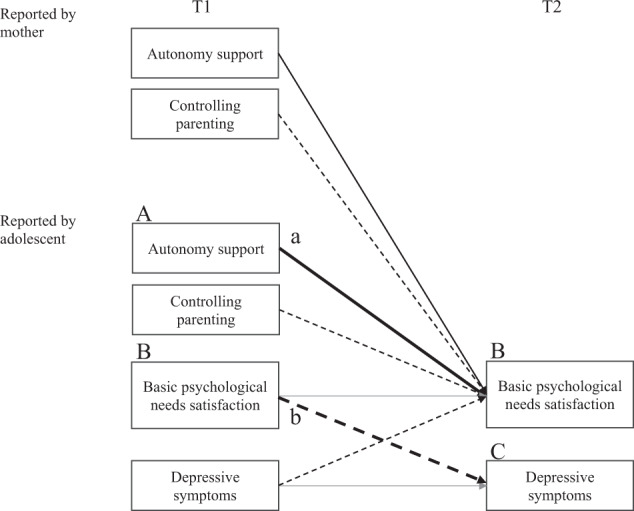
The model explored in the present study. Under the stationarity assumption, for example, if paths a and b are significant, the paths would provide the estimate of the mediational effect of the relation between A and C by B (Cole & Maxwell, 2003; for application, see Ohtani et al., 2020). Dotted lines indicate that the relations are hypothesized to be negative

Statistical analyses were carried out using R version 4.0.0 (2020-04-24) and the preregistered analysis plan (https://osf.io/2eaz7) was followed with some modification mentioned earlier. To examine the main hypotheses, two-wave structural cross-lagged models were formulated (Fig. 1). Unlike the most common type of mediational study utilizing a cross-sectional approach, which could generate substantial biases (Maxwell & Cole, 2007; 2011), a longitudinal design enabled us to consider autoregressive effects and time lags. Potential mediational processes can be tested under certain assumptions, when two waves of data are available (Cole & Maxwell, 2003). One is the assumption of stationarity: the causal structure remains the same over time, and the other assumption is that optimal time lag for the independent variable to affect the mediator is the same as the time lag for the mediator to affect the dependent variable. For example, if paths a and b in Fig. 1 are statistically significant, their product would provide an estimate of the mediational effect of the relation between A and C by B (for an example of application, see Ohtani et al., 2020). The stationarity and optimal time lag assumptions are not testable with the two waves of data; however, it is considered acceptable for this present study to be based on the assumptions considering the relatively short four-month time-lag design. To test the mediational effect (i.e., product of paths a and b), the Monte Carlo method was implemented to obtain an empirical sampling distribution of estimated parameters of effects and robust confidence intervals, using the R lavaan and semTools packages (MacKinnon et al., 2004).

To address missing data in the main analyses^3^, multiple imputation was performed by implementing bootstrapping based expectation-maximization (EM) algorithm using the Amelia package in R (Honaker et al., 2011). The imputation was performed using all the variables, including demographic variables used in the subsequent analyses and parent–child relationship satisfaction reported by the mothers and adolescents. A total of 500 imputed data sets were created and the results were aggregated.

Structural equation modeling with observed variables was applied and may be preferable to the one with latent variables in terms of the simplicity and accuracy of estimation given the modest sample size (Ledgerwood & Shrout, 2011; Savalei, 2018). However, it assumes all measures have perfect reliability. As supplemental analysis, a structural equation modeling with fixed reliability single indicator model (Savalei, 2018) was implemented to control for measurement error in a simple way.

To judge the significance of the beta values, the standard 0.05 threshold for *p*-values was used. Unstandardized regression coefficients (B), their 95% confidence intervals, and standardized beta coefficients (*β*) were reported.

## Results

### Means, Standard Deviations, and Correlations Among Variables: Mother–child Agreement for Parenting Behavior

Table 1 presents the means and standard errors of the variables at T1 and T2. Skewness and kurtosis were examined for all variables and indicated that they were within an absolute skew value of 2 and absolute kurtosis value of 3 at both T1 and T2, suggesting a normal distribution of variables based on the sample size (*n* > 300; Kim, 2013).

**Table 1:** Descriptive statistics of all variables at T1 and T2

Variables	*Possible range*	T1						T2					
		*N*	*M*	*SE*	*Observed range*	*Skewness*	*Kurtosis*	*N*	*M*	*SE*	*Observed range*	*Skewness*	*Kurtosis*
Mother’s self-report													
Autonomy support	1.00–7.00	407	4.95	0.03	2.75–7.00	0.35	0.45	369	4.98	0.04	2.50–6.83	−0.08	0.32
Controlling parenting	1.00–7.00	405	2.51	0.04	1.00–6.08	0.35	−0.36	371	2.44	0.05	1.00–6.58	0.54	−0.03
Adolescents’ self-report													
Perceived Autonomy support	1.00–7.00	404	5.14	0.05	1.42–7.00	−0.31	−0.13	367	5.25	0.06	1.33–7.00	−0.38	−0.17
Perceived Controlling parenting	1.00–7.00	397	2.23	0.05	1.00–7.00	1.22	1.71	364	2.17	0.06	1.00–7.00	1.29	1.59
Basic psychological needs satisfaction	1.00–5.00	403	3.49	0.03	1.00–5.00	−0.26	0.66	371	3.56	0.04	1.33–5.00	−0.20	0.35
Depressive symptoms	1.00–3.00	406	1.59	0.02	1.00–2.78	0.67	0.31	371	1.58	0.02	1.00–2.89	0.65	0.31

*N* number of participants, *M* mean, *SE* standard error

Table 2 shows the correlations between all variables. Consistent with the Hypotheses 1, mother–child agreement (i.e., correlations between mothers’ self-reports and adolescents’ perception) for autonomy support and controlling parenting ranged from 0.27 to 0.39^4^. Neither mothers’ and adolescents’ ages nor family socioeconomic status showed a significant relation with other variables. Adolescents’ sex was statistically significantly related with adolescents’ perceived controlling parenting at T2.

**Table 2:** Correlations among all variables

	T1						T2								
	Reported by mothers		Reported by adolescents				Reported by mothers		Reported by adolescents						
	1	2	3	4	5	6	7	8	9	10	11	12	13	14	15
T1															
Reported by mothers															
1. Autonomy support	⎯														
2. Controlling parenting	−0.46*	⎯													
Reported by adolescents															
3. Perceived autonomy support	0.35*	−0.21*	⎯												
4. Perceived control parenting	−0.22*	0.33*	−0.52*	⎯											
5. Basic psychological needs satisfaction	0.12	−0.05	0.49*	−0.31*	⎯										
6. Depressive symptoms	−0.13	0.08	−0.32*	0.24*	−0.62*	⎯									
T2															
Reported by mothers															
7. Autonomy support	0.76*	−0.43*	0.35*	−0.23*	0.19*	−0.15	⎯								
8. Controlling parenting	−0.40*	0.76*	0.19*	0.30*	−0.05	0.08	−0.45*	⎯							
Reported by adolescents															
9. Perceived autonomy support	0.32*	−0.18	0.75*	−0.43*	0.46*	−0.31*	0.39*	−0.16	⎯						
10. Perceived control parenting	−0.22*	0.24*	−0.42*	0.65*	−0.24*	0.18	−0.19*	0.27*	−0.49*	⎯					
11. Basic psychological needs satisfaction	0.19*	−0.04	0.41*	−0.31*	0.71*	−0.51*	0.27*	−0.06	0.56*	−0.30*	⎯				
12. Depressive symptoms	−0.16	0.09	−0.31*	0.30*	−0.52*	0.66*	−0.21*	0.09	−0.42*	0.34*	−0.63*	⎯			
Demographic Variables															
13. Adolescents’ sex (Male = 0, Female = 1)	−0.04	−0.14	0.08	−0.15	0.07	0.10	0.07	−0.15	0.13	−0.20*	0.05	0.01	⎯		
14. Adolescents’ age	0.04	−0.06	0.06	−0.10	0.05	0.02	0.03	−0.06	0.11	−0.06	0.07	−0.01	0.07	⎯	
15. Mothers’ age	0.06	−0.07	−0.06	−0.01	0.03	−0.01	0.13	−0.10	−0.03	0.00	0.01	−0.02	0.06	0.12	⎯
16. Family socioeconomic status	−0.06	0.00	−0.07	−0.02	0.07	−0.10	−0.01	0.01	−0.05	−0.02	0.08	−0.13	0.05	0.10	0.04

Underlined correlations show mother–child agreement. For family socioeconomic status, Spearman’s rank-order correlation coefficients were used

*p < 0.05 (adjusted for multiple tests)

### Associations between Parenting Behavior Reported by Mothers and Adolescents and Depressive Symptoms: Mediation of Basic Psychological Needs Satisfaction

**Fig. 2:**
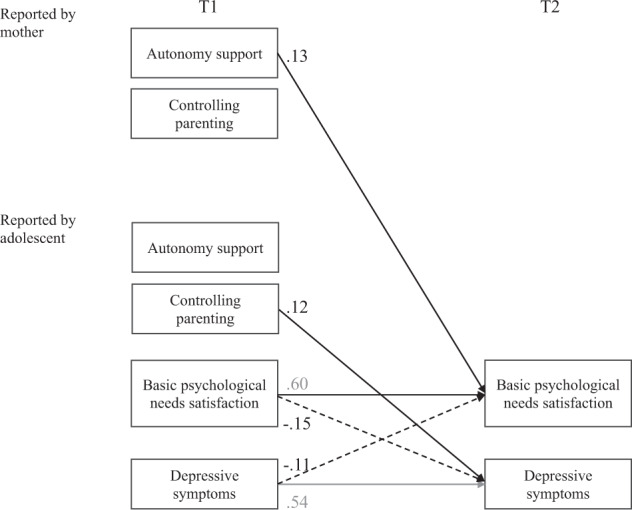
Standardized coefficients from regression models. Non-significant paths (*p* ≥ 0.05) were omitted from the figure for brevity. Dotted lines indicate that the relations were negative

Table 3 presents all results of the structural cross-lagged regression analyses; the statistically significant results are summarized in Fig. 2. Adolescents’ basic psychological needs satisfaction and depressive symptoms were regressed on autonomy support and controlling parenting reported by mothers and perceived by adolescents, adolescents’ basic psychological needs satisfaction, and depressive symptoms, all measured at T1. Adolescents’ sex was excluded from the model because there was no significant relationship with the dependent variables, and the results were same regardless of its inclusion.

**Table 3:** Predictions of basic psychological needs satisfaction and depressive symptoms

Predictor variable (T1)	(T2)							
	Basic psychological needs satisfaction				Depressive symptoms			
	*R*^2^ = 0.52				*R*^2^ = 0.47			
	*B* [95%CI]	*SE B*	β	*p*	*B* [95%CI]	*SE B*	β	*p*
Reported by mothers								
Autonomy support	0.14 [0.05, 22]	0.05	0.13	<0.01	−0.04 [−0.08, 0.00]	0.02	−0.07	0.07
Controlling parenting	0.07 [−0.01, 0.14]	0.04	0.08	0.07	−0.01 [−0.04, 0.03]	0.02	−0.02	0.66
Reported by adolescents								
Perceived autonomy support	0.00 [−0.07, 0.07]	0.03	0.00	0.99	0.01 [−0.03, 0.04]	0.02	0.02	0.73
Perceived control parenting	−0.06 [−0.13, 0.00]	0.03	−0.09	0.06	0.04 [0.01, 0.08]	0.02	0.12	0.02
Basic psychological needs satisfaction	0.62 [0.52, 0.73]	0.05	0.60	<0.01	−0.08 [−0.14, −0.02]	0.03	−0.15	<0.01
Depressive symptoms	−0.21 [−0.39, −0.03]	0.09	−0.11	0.02	0.55 [0.45, 0.65]	0.05	0.54	<0.01

As shown in Table 3 and Fig. 2, mothers’ reported autonomy support at T1 positively predicted the change in adolescents’ basic psychological needs satisfaction: the more mothers reported autonomy support at T1, the more adolescents’ basic psychological needs satisfaction increased from T1 to T2. Contrary to Hypotheses 2, adolescents’ perceived autonomy support and controlling parenting showed no significant relation with their basic psychological needs satisfaction. Consistent with Hypotheses 3, basic psychological needs satisfaction negatively predicted the change in adolescents’ depressive symptoms. The more satisfied they felt with their basic psychological needs at T1, the more their depressive symptoms decreased from T1 to T2. Adolescents’ perceived controlling parenting also negatively predicted depressive symptoms.

The significance of the mediation of adolescents’ basic psychological needs satisfaction on the relation between mothers’ reported autonomy support and adolescents’ depressive symptoms was then tested. The mediational effects demonstrated that they were statistically significant for mothers’ reported autonomy support (−0.01, 95%CI [−0.02, −0.002]).

Additionally, adolescents’ depressive symptoms significantly predicted their basic psychological needs satisfaction. The more adolescents reported depressive symptoms at T1, the more their basic psychological needs satisfaction decreased from T1 to T2.

### Supplemental Analysis

To the supplement the main analysis, latent variable structural equation modeling with fixed reliability single indicator model was implemented. In the model, each latent variable was represented by a scale score. The reliability coefficient of each construct was fixed to 0.80, following the recommendation by Savalei (2018)^5^. To solve the convergence problem, the autoregression coefficients of basic psychological needs satisfaction and depressive symptoms were fixed at 0.60 and 0.55, respectively. The pattern of the results was nearly identical to the one obtained above (see Appendix, Table 7). A statistically significant mediational effect of adolescents’ basic psychological needs satisfaction was obtained for the relationship between mothers’ reported autonomy support and adolescents’ depressive symptoms (−0.02, 95%CI [−0.04, −0.001]). The results from the alternative analytical approaches indicate the robustness of the main findings.

## Discussion

As indicated by numerous studies on self-determination theory, parental autonomy support versus controlling parenting can predict mental wellness among adolescents (Ryan & Deci, 2017). However, researchers have paid relatively little attention to incongruence in mother-reported and adolescent-perceived autonomy support and controlling parenting, and no studies have examined their possible independent roles on adolescents’ basic psychological needs satisfaction. The present study aimed to shed light on incongruence between mothers’ report and adolescent children’s perception and examine their possible independent roles on adolescents’ basic psychological needs satisfaction, which is supposed to link the impact of parenting on adolescents’ well-being outcomes (Ryan & Deci, 2017). The analyses confirmed low to moderate agreement and revealed statistically significant mediation by basic psychological needs satisfaction for the relationship between mothers’ reported, but not adolescents’ perceived, autonomy support and depressive symptoms in adolescents.

### Associations between Parenting Behavior Reported by Adolescents and Mothers

All four mother–child agreements were low or moderate at maximum. Results consistent with past evidence (Cheung et al., 2016; Korelitz & Garber, 2016; Nelemans et al., 2020; Vrolijk et al., 2020) were obtained for the first time in a sample of Japanese mother–child dyads. Incongruence between parents’ and children’s reports might be an inescapable consequence of differing points of view (Edelbrock et al., 1986; Tein et al., 1994). Adolescence is a time of changing family relationships and is characterized by discrepancies between parents and children (Pelegrina et al., 2003). The present results showed that adolescents’ perception did not necessarily reflect mother-reported parenting behavior. Researchers must be cautious when assessing parental behaviors and keep in mind the discordance to avoid inaccurate conclusions. Future research should explore factors that directly contribute to the formation of adolescents’ own perspective of autonomy support and controlling parenting.

### Associations between Parenting Behavior Reported by Mothers and Adolescents and Depressive Symptoms: Mediation of Basic Psychological Needs Satisfaction

It was hypothesized that autonomy support and controlling parenting perceived by adolescents would positively and negatively predict basic psychological needs satisfaction, respectively, and that a higher level of adolescents’ basic psychological needs satisfaction would lead to a lower level of depressive symptoms. Consistent with the hypothesis, the results showed that adolescents’ basic psychological needs satisfaction negatively predicted depressive symptoms. However, mothers’ reported autonomy support, rather than that perceived by adolescents, positively predicted basic psychological needs satisfaction. This finding contradicted what was expected in light of previous research. In the present study, the parenting variables of both reporters were included in the same regression analyses, a different approach from that in most previous studies (Janssens et al., 2015; Nelemans et al., 2020; Paulson, 1994; Vrolijk et al., 2020). The results provide insight into the independent predictive capacity of mothers’ reports and adolescents’ perceptions in the following three aspects.

First, autonomy support reported by mothers was significantly associated with adolescents’ basic psychological needs satisfaction, even after autonomy support reported by adolescents was added to the regression model. Thus, maternal autonomy support seems to be beneficial regardless of how adolescents perceived it. The effect size was not large (*B* = 0.14), but the association is important given that the relation between the variables measured by adolescents’ own perspectives can be typically over-estimated due to the lack of independence of the data. The present results would justify mothers’ own efforts to provide autonomy support. As with the large body of parental training programs (see Kaminski et al., 2008), autonomy-supportive methods can be acquired and taught. Despite the accumulated empirical evidence and its potential, work is only beginning in this area (Allen et al., 2019). For example, Joussemet et al. (2014) implemented and evaluated the effect of a program called “How to talk so kids will listen and listen so kids will talk” to promote autonomy-supportive approaches to parents. The program includes sessions on how to listen to and acknowledge the child, encourage the child’s initiatives, and help them achieve their full potential. It was found to be effective in improving parenting style and promoting children’s mental health. Further research will be needed to develop and promote training programs.

Second, adolescents’ perception of controlling parenting was associated positively with depressive symptoms. Particular attention should be given to the result that perceived controlling parenting, more precisely, its residuals above and beyond mothers’ reported controlling parenting, might be one of the critical risk factors of depressive symptoms. Self-determination theory suggests that the more controlling the parenting, the more the adolescents experienced that they are not responsible for their action, and considerations of options that would be more congruent with their needs and interests are often precluded (Deci & Ryan, 1987; Ryan et al., 1997). It is important for mothers to know that their adolescents may be negatively affected by perception of their controlling parenting, which their adolescents perceive differently than they perceive themselves. Furthermore, as incorporated in some depression prevention programs using cognitive-behavioral interventions (e.g., Gillham et al., 2006), it would also be efficacious to teach adolescents the skills to be assertive and negotiate with their mother when they perceive mothers’ behavior as controlling. More research is needed to investigate what contributes to adolescents’ perceptions of controlling parenting.

Third, the present results replicated the direct protective effect of basic psychological needs satisfaction against depressive symptoms in adolescence (e.g., Costa et al., 2016). The effect size is comparable to that of other research and intervention efforts on depression (see Cairns et al., 2014, for a meta-analysis). As Ryan (1992, p.5) argued, when an activity is experienced as stemming from the self, it is experienced as vital: “this vitality is exuded because individuals operate from the energetic center of animate existence and thus tap the springs of their own living nature.” If this vitality is lost, mental problems are likely to emerge in adolescents. It was also found that the relation is reciprocal: if depressive symptoms emerge, adolescents tend to experience less satisfaction regarding their basic needs, which would lead to a further decline in their vitality. The present study demonstrated that for those with depressive symptoms, support for satisfaction of their basic needs, namely, autonomy, competence, and relatedness, would be of great significance.

### Limitations and Future Research Directions

The limitations of the present study should be noted. First, this study relied only on the self-reported data of each informant. Although what mothers think they are doing and what adolescents think their mothers are doing might be valuable subjective realities to assess and address as they are, the assessment of maternal behavior in the home as observed by the father or another family member is a promising method that can provide an external criterion against which mothers’ and adolescents’ reports can be measured. What mothers think about how adolescents perceive their parenting (Mageau et al., 2017) also merits investigation.

Second, participants completed only two assessments, and when the mediation was tested, both the mediator and outcome variables were located at the second time point. Although it is reasonable to think that stationarity assumption of the causal structure can be applied in the present study, three assessments or more would be necessary in future studies to examine the mediation effect.

Third, the present study looked only at maternal autonomy support, not paternal support. Findings are inconsistent on whether maternal and paternal autonomy support and controlling parenting are equally importance (Duineveld et al., 2017; Van der Giessen, 2014) or not (Costa et al., 2019) during adolescence. Whether the present results on the level of agreement between the parenting behavior reported by mothers and adolescents and their relative influences on basic psychological needs and depressive symptoms are replicated with paternal parenting remains an open question for future research.

Finally, many studies using self-determination theory have shown similar influences of autonomy support and controlling parenting across different cultures (e.g., Chen et al., 2015; Soenens et al., 2012). The present study, conducted in a cultural context characterized by low satisfaction and high anxiety regarding their parenting for mothers, who have the strong central responsibility for caregiving and socialization of adolescents (Kayama, 2010; NWEC, 2006), well-contributes to this perspective. However, the present findings may not be generalizable to other cultures. Directions for future research should include cross-cultural investigations of parent–child agreement and its relative importance on adolescents’ well-being.

## Conclusion

Most studies on autonomy support and controlling parenting using self-determination theory have relied on children’s perceptions, ignoring findings from multi-informant studies that highlight the limits of this approach. Moreover, there is a lack of research examining the relative impact of autonomy support and controlling parenting reported by parents and adolescents on basic psychological needs satisfaction of adolescents. The present study confirmed that adolescents may perceive parenting differently than their mothers do. Regardless of adolescents’ perceptions, maternal autonomy support might be beneficial; regardless of mothers’ report, adolescents’ perceived controlling parenting is damaging for adolescents’ mental health. Further research on unraveling this complex phenomenon and providing effective support for adolescents’ growth is essential.

### Supplementary information

Supplementary Information

## Appendix

Tables 4–7

**Table 4:** Distribution of family socioeconomic status (income) (unit: million JPY)

		*N*	%
1.	<2	14	3.5
2.	2⎯2.99	25	6.2
3.	3⎯3.99	34	8.4
4.	4⎯4.99	31	7.7
5.	5⎯5.99	64	15.8
6.	6⎯6.99	63	15.6
7.	7⎯7.99	57	14.1
8.	8⎯8.99	33	8.2
9.	9⎯9.99	31	7.7
10.	10⎯11.99	30	7.4
11.	12⎯14.99	17	4.2
12.	>15	5	1.2
Total		404	
Missing		4

**Table 5:** Factor loadings with oblimin rotation for perceived parental autonomy support scale (P-PASS) reported by adolescents, eigenvalues, and percentages of explained variance for each factor

Item		*Factor loading*	
		1	2
Autonomy Support			
Choice Within Certain Limits			
1.	My mother gave me many opportunities to make my own decisions about what I was doing. *お母さんは, 私が自分ですることを自分で決める機会をたくさんくれた*	0.66	−0.16
4.	My point of view was very important to my mother when she made important decisions concerning me. *お母さんが私のことに関する何か重要な決断をする時, 私の考えを大事にしてくれた*	0.75	0.08
8.	Within certain limits, my mother allowed me the freedom to choose my own activities *お母さんは, 私が自分の行動を自分で決める自由をある程度の範囲で与えてくれた*	0.59	−0.14
14.	My mother hoped that I would make choices that corresponded to my interests and preferences regardless of what hers were. *お母さんは, 私がお母さんの興味や関心に関係なく, 自分の興味や関心で選択を行うことを望んだ*	0.48	−0.03
Rationale for Demands and Limits			
2.	When my mother asked me to do something, she explained why she wanted me to do it. *お母さんが私に何かして欲しいと頼んだときは, その理由を説明してくれた*	0.68	−0.01
9.	When I was not allowed to do something, I usually knew why. *お母さんが私のしようとしたことを禁止した時は, たいていどうして禁止されたのか分かった*	0.57	0.02
19.	My mother made sure that I understood why she forbid certain things. *お母さんは, 私に対して何かを禁止する時には私がその理由を確実に理解できるようにした*	0.67	0.08
23.	When I asked why I had to do, or not do, something, my mother gave me good reasons. *やらなければいけないこと, またはやってはいけないことがあった時, なぜそれに従わなくてはいけないのか, とお母さんに聞くと, 私に納得できる理由を説明してくれた*	0.73	0.03
Acknowledgement of Feelings			
7.	My mother encouraged me to be myself. *お母さんは, 私が私自身でいられるよう励ましてくれた*	0.78	0.05
13.	My mother was able to put herself in my shoes and understand my feelings. *私のお母さんは, 私の立場で考え, 私の気持ちを理解してくれた*	0.85	0.02
16.	My mother was open to my thoughts and feelings even when they were different from hers. *お母さんは, たとえ私の考えや気持ちが自分と違っていても, 私の立場を理解した態度を示してくれた*	0.80	0.02
24.	My mother listened to my opinion and point of view when I disagreed with her. *私がお母さんと対立してしまったとき, お母さんは私の意見や考え方を聞いてくれた*	0.76	0.00
Controlling Parenting			
Threats to Punish			
3.	When I refused to do something, my mother threatened to take away certain privileges in order to make me do it. *私がお母さんの望む通りのことをしないと, お母さんはそれをさせるために, 私への接し方を悪くすると脅した*	−0.09	0.65
10.	I always had to do what my mother wanted me to do, if not, she would threaten to take away privileges. *私はいつもお母さんが望む通りのことをしなくてはいなかった。さもないとお母さんは, 私への接し方を悪くすると脅してきたからだ*	−0.06	0.80
15.	When my mother wanted me to do something, I had to obey or else I was punished. *お母さんが私に何かをしてほしいと望むとき, 私はそれに従わなければならず, そうしなければ, お仕置きをされた*	−0.01	0.82
20.	As soon as I didn’t do exactly what my mother wanted, she threatened to punish me. *私のしたことがお母さんが望むことと少しでも違っていたとわかると, お母さんはお仕置きをすると脅してきた*	0.06	0.90
Performance Pressures			
5.	My mother refused to accept that I could want simply to have fun without trying to be the best. *1番になろうとはせず, 単に楽しみたいということをお母さんは受け入れてくれなかった*	−0.29	0.39
11.	My mother believed that, in order to succeed, I always had to be the best at what I did. *お母さんは, 私が成功するために, 自分のすることでいつも1番になるべきだと考えていた*	0.12	0.41
17.	In order for my mother to be proud of me, I had to be the best. *お母さんが私に誇りを持つために, 私は1番にならなければいけなかった*	0.01	0.63
22.	My mother insisted that I always be better than others. *常に他人よりも優れていることをお母さんは私に要求した*	−0.06	0.61
Guilt-Inducing Criticisms			
6.	When my mother wanted me to do something differently, she made me feel guilty. *お母さんが望むような行動ができないとき, 私は罪悪感を感じさせられた*	0.01	0.53
12.	My mother made me feel guilty for anything and everything. *お母さんのせいで私はすべてのことについて後ろめたく感じた*	−0.09	0.73
18.	When my mother wanted me to act differently, she made me feel ashamed in order to make me change. *お母さんは私に恥ずかしさを感じさせることで, 私の振る舞いを変えようとした*	−0.01	0.63
21.	My mother used guilt to control me. *お母さんは私をコントロールするために罪悪感を利用した*	0.06	0.91
Eigenvalue		9.86	3.17
% of variance		0.26	0.25

**Table 6:** Factor loadings with oblimin rotation for perceived parental autonomy support scale (P-PASS) reported by mothers, eigenvalues, and percentages of explained variance for each factor

Item		*Factor loading*	
		1	2
Autonomy Support			
Choice Within Certain Limits			
1.	I gave my child many opportunities to make his/her own decisions about what he/she was doing. *私は, 子どもが自分ですることを自分で決める機会をたくさん与えた*	−0.20	0.43
4.	My child’s point of view was very important to me when I made important decisions concerning him/her. *私が子どものことに関する何か重要な決断をする時, 私は子どもの考えを大事にした*	−0.18	0.55
8.	Within certain limits, I allowed my child the freedom to choose his/her own activities *私は子どもが自分の行動を自分で決める自由をある程度の範囲で与えた*	−0.20	0.52
14.	I hoped that my child would make choices that corresponded to his/her interests and preferences regardless of what mine were. *子どもが私の興味や関心に関係なく, 自分の興味や関心で選択を行うことを望んだ*	−0.02	0.28
Rationale for Demands and Limits			
2.	When I asked my child to do something, I explained why I wanted him/her to do it. *私が子どもに何かして欲しいと頼んだときは, その理由を説明した*	0.12	0.67
9.	When I did not allow my child to do something, he/she usually knew why. *私が子どものしようとしたことを禁止した時は, たいていどうして禁止したのか子どもに理解させた*	0.11	0.67
19.	I made sure that my child understood why I forbid certain things. *私は, 子どもに対して何かを禁止する時には子どもがその理由を確実に理解できるようにした*	0.11	0.70
23.	When my child asked why he/she had to do, or not do, something, I gave him/her good reasons. *子どもがやらなければいけないこと, またはやってはいけないことがあった時, なぜそれに従わなくてはいけないのか, と子どもに聞かれると, 私は子どもに納得できる理由を説明した*	0.10	0.71
Acknowledgement of Feelings			
7.	I encouraged my child to be himself/herself. *私は, 子どもが自分自身でいられるよう励ました*	−0.10	0.58
13.	I was able to put myself in my child’s shoes and understand his/her feelings. *私は, 子どもの立場で考え, 子どもの気持ちを理解した*	−0.21	0.57
16.	I was open to my child’s thoughts and feelings even when they were different from mine. *私は, たとえ子どもの考えや気持ちが自分と違っていても, 子どもの立場を理解した態度を示した*	−0.30	0.47
24.	I listened to my child’s opinion and point of view when he/she disagreed with me. *子どもが私と対立してしまったとき, 私は子どもの意見や考え方を聞いた*	−0.12	0.60
Controlling Parenting			
Threats to Punish			
3.	When my child refused to do something, I threatened to take away certain privileges in order to make him/her do it. *子どもが私の望む通りのことをしないと, 私はそれをさせるために, 子どもへの接し方を悪くすると脅した*	0.58	−0.09
10.	My child always had to do what I wanted him/her to do, if not, I would threaten to take away privileges. *子どもはいつも私が望む通りのことをしなくてはいなかった。さもないと私は, 子どもへの接し方を悪くすると脅してきたからだ*	0.80	−0.03
15.	When I wanted my child to do something, he/she had to obey or else he/she was punished. *私が子どもに何かをしてほしいと望むとき, 子どもはそれに従わなければならず, そうしなければ, お仕置きをした*	0.68	−0.08
20.	As soon as my child didn’t do exactly what I wanted, I threatened to punish him/her. *子どものしたことが私が望むことと少しでも違っていたとわかると, 子どもにお仕置きをすると脅した*	0.78	−0.03
Performance Pressures			
5.	I refused to accept that my child could want simply to have fun without trying to be the best. *子どもが1番になろうとはせず, 単に楽しみたいということを私は受け入れなかった*	0.51	−0.15
11.	I believed that, in order to succeed, my child always had to be the best at what he/she did. *子どもは成功するために, 自分のすることでいつも1番になるべきだと私は考えていた*	0.57	0.11
17.	In order for me to be proud of my child, he/she had to be the best. *私が子どもに誇りを持つために, 子どもは1番にならなければいけなかった*	0.60	0.04
22.	I insisted that my child always be better than others. *常に他人よりも優れていることを私は子どもに要求した*	0.69	0.07
Guilt-Inducing Criticisms			
6.	When I wanted my child to do something differently, I made him/her feel guilty. *私が望むような行動ができないとき, 子どもに罪悪感を感じさせた*	0.68	−0.03
12.	I made my child feel guilty for anything and everything. *子どもにすべてのことについて後ろめたく感じさせた*	0.62	−0.15
18.	When I wanted my child to act differently, I made him/her feel ashamed in order to make him/her change. *私は子どもに恥ずかしさを感じさせることで, 子どもの振る舞いを変えようとした*	0.66	0.10
21.	I used guilt to control my child. *私は子どもをコントロールするために罪悪感を利用した*	0.81	0.05
Eigenvalue		8.30	3.05
% of variance		0.25	0.18

**Table 7:** Predictions of basic psychological needs satisfaction and depressive symptoms

Predictor variable (T1)	(T2)							
	Basic psychological needs satisfaction				Depressive symptoms			
	*R*^2^ = 0.52				*R*^2^ = 0.47			
	*B* [95%CI]	*SE B*	β	*p*	*B* [95%CI]	*SE B*	β	*p*
Reported by mothers								
Autonomy support	0.20 [0.03, 37]	0.09	0.19	0.02	−0.07 [−0.15, 0.01]	0.04	−0.13	0.07
Controlling parenting	0.12 [−0.01, 0.25]	0.07	0.16	0.07	−0.03 [−0.09, 0.03]	0.03	−0.08	0.34
Reported by adolescents								
Perceived autonomy support	0.01 [−0.11, 0.12]	0.06	0.01	0.94	0.03 [−0.05, 0.11]	0.04	0.09	0.44
Perceived control parenting	−0.09 [−0.20, 0.03]	0.06	−0.14	0.15	0.07 [0.00, 0.13]	0.03	0.21	0.04
Basic psychological needs satisfaction	0.60^a^	–	0.62	–	−0.09 [−0.17, −0.02]	0.04	−0.18	0.02
Depressive symptoms	−0.23 [−0.42, 0.03]	0.10	−0.12	0.02	0.55^a^	–	0.59	–

χ^2^(2) = 30.11, *p* < 0.01, CFI = 0.98, SRMR = 0.03, RMSEA = 0.19

^a^Fixed coefficients were used

## Footnotes

1 Aligning with the suggestions from multiple reviewers, the pre-registered analyses regarding the longitudinal prediction of adolescents’ perceived parenting by mothers’ reported parenting were removed from the main text and Hypotheses 1 was modified. Those results are presented in the supplemental materials.

2 The change was made in accordance with the suggestions from multiple reviewers. The results from the analyses for that included the mother–child relationship satisfaction as a control variable are presented in the supplemental materials.

3 The attrition effect was examined in the T1 variables between participants who missed at T2 (*N* = 35) and those who participated (*N* = 373). For mothers’ self-report, no significant mean differences in all variables between the two groups was found, including for T1 autonomy support, *t* (405) = 1.04, *p* = 0.30, Hedges *g* = 0.18, and controlling parenting, *t* (403) = 0.99, *p* = 0.32, *g* = 0.17. For adolescents’ self-report, there was no significant mean difference for all variables, including perception of autonomy support, *t* (402) = 1.93, *p* = 0.05, *g* = 0.34, perception of controlling parenting, *t* (395) = 0.95, *p* = 0.34, *g* = 0.17, basic psychological needs satisfaction, *t* (401) = 0.66, *p* = 0.51, *g* = 0.12, and depressive symptoms, *t* (404) = 1.66, *p* = 0.099, *g* = 0.30.

4 There was no difference in mother-child agreement by sex, family socio-economic status, and depressive symptoms. Significant age difference was found: Mother and younger adolescent pairs showed lower agreement than mother and older adolescents. The results are available upon request.

5 Similar results were produced when reliability was fixed to Cronbach’s alpha of the scale.
