# Supplementary material for: Longitudinal Association between Maternal Autonomy Support and Controlling Parenting and Adolescents’ Depressive Symptoms
Source: J Youth Adolesc. 2023 Jan 19;52(5):1058–73. doi: 10.1007/s10964-022-01722-1 (PMC9851735; doi:10.1007/s10964-022-01722-1)
Supplement: Supplementary file 1 — Supplementary Information [file 10964_2022_1722_MOESM1_ESM.docx]

**Supplementary Materials for**

Longitudinal Association between Maternal Autonomy Support and Controlling Parenting and Adolescents’ Depressive Symptoms

**Table of Contents**

Description of Contents [1](#_Toc100235696)

Predictions of Autonomy Support and Controlling Parenting [2](#_Toc100235696)

Predictions of Basic Psychological Needs Satisfaction and Depressive Symptoms [5](#_Toc100235696)

References  [8](#_Toc100235696)

**Description of Contents**

This document contains the supplementary analyses referenced in the main manuscript. First, we present the results of the cross-lagged model regarding longitudinal predictions of autonomy support and controlling parenting, both for that reported by mothers and perceived by adolescents. Second, we present the results of the main analyses conducted with additional control variables : Parent–child relationship satisfaction reported by mothers and adolescents, and demographic variables.

#

**Predictions of Autonomy Support and Controlling Parenting**

We examined the longitudinal association between adolescents’ perceptions of parenting behaviors and the parenting behaviors reported by their mothers. Specifically, we investigated if the adolescents’ perceptions of parenting measured at T2 were predicted by mothers’ reported parenting (autonomy support and controlling parenting) at T1, above and beyond the influence of adolescents’ perceptions of parenting measured at T1. In a similar manner, we also investigated if changes of mothers’ reported parenting from T1 to T2 were predicted by adolescents’ reported parenting at T1. For these analyses, we controlled for the effect of adolescents’ depressive symptoms and basic psychological needs satisfaction measured at T1.

Based on the conventional perspective, we expected adolescents’ perceptions of maternal parenting behavior to be predicted by mothers’ reported parenting behavior, but that relations may not be strong. Specifically, we hypothesized that autonomy support reported by adolescents would be predicted positively and negatively by the autonomy support and controlling parenting reported by mothers, respectively. Controlling parenting reported by adolescents would be predicted negatively and positively by the autonomy support and controlling parenting reported by mothers, respectively (Hypotheses 1 in the pre-registration).

Table S1 presents the results of cross-lagged regression analyses regarding the prediction of adolescents’ perceived parenting. Our hypotheses were partially supported. Mothers’ reported autonomy support at T1 was significantly related with the change of adolescents’ perceived autonomy support from T1 to T2. This implies mothers’ reported autonomy supporting behavior at T1 would be reflected in adolescents’ perception as it is. In contrast, there was no significant relationship for controlling parenting among mothers’ report and adolescents’ perception. This implies the possibility that adolescents perceive mothers’ controlling parenting in a different way than mothers think about their way of controlling parenting.

| Table S1.  *Predictions of Adolescents’ Perceived Autonomy Support and Controlling Parenting* | | | | | | | | | |
| --- | --- | --- | --- | --- | --- | --- | --- | --- | --- |
|  | Perceived Autonomy Support  (T2) | | | |  | Perceived Controlling Parenting  (T2) | | | |
|  | *R*^2^ = .58 | | | |  | *R*^2^ = .44 | | | |
| Predictor variable (T1) | *B* [95%CI] | *SE B* | β | *p* |  | *B* [95%CI] | *SE B* | β | *p* |
| Reported by mothers |  |  |  |  |  |  |  |  |  |
| Autonomy support | .13 [.01, .26] | .06 | .08 | .03 |  | -.08 [-.24, .09] | .08 | -.04 | .35 |
| Controlling  parenting | -.00 [-.09, .09] | .05 | -.00 | .98 |  | .02 [-.11, .14] | .06 | .01 | .77 |
| Reported by adolescents |  |  |  |  |  |  |  |  |  |
| Perceived autonomy  support | .69 [.59, .79] | .05 | .66 | <.00 |  | -.12 [-.24, .01] | .06 | -.11 | .06 |
| Perceived control  parenting | -.05 [-.14, .05] | .05 | -.05 | .33 |  | .64 [.48, .80] | .08 | .59 | <.00 |
| Basic psychological needs satisfaction | .13 [-.03, .28] | .08 | .08 | .10 |  | -.01 [-.19, .18] | .09 | -.01 | .93 |
| Depressive  symptoms | -.08 [-.34, .18] | .13 | -.02 | .53 |  | -.06 [-.37, .26] | .16 | -.02 | .72 |

| Table S2.  *Predictions of Mothers’ Reported Autonomy Support and Controlling Parenting* | | | | | | | | | |
| --- | --- | --- | --- | --- | --- | --- | --- | --- | --- |
|  | Autonomy Support (T2) | | | |  | Controlling Parenting (T2) | | | |
|  | *R*^2^ = .60 | | | |  | *R*^2^ = .59 | | | |
| Predictor variable (T1) | *B* [95%CI] | *SE B* | β | *p* |  | *B* [95%CI] | *SE B* | β | *p* |
| Reported by mothers |  |  |  |  |  |  |  |  |  |
| Autonomy support | .71 [.63, .79] | .05 | .13 | <.01 |  | -.10 [-.22, .03] | .06 | -.10 | .12 |
| Controlling  parenting | -.07 [-.14, -.01] | .04 | .08 | .04 |  | .78 [.68, .87] | .05 | .78 | .00 |
| Reported by  adolescents |  |  |  |  |  |  |  |  |  |
| Perceived autonomy  support | .04 [-.02, .10] | .03 | .00 | .21 |  | .01 [-.08, .09] | .04 | .01 | .90 |
| Perceived control  parenting | .00 [-.05, .06] | .03 | -.09 | .94 |  | .07 [-.01, .15] | .04 | .07 | .08 |
| Basic psychological  needs satisfaction | .09 [.01, .18] | .05 | .60 | .04 |  | .02 [-.12, .16] | .07 | .02 | .77 |
| Depressive  symptoms | .03 [-.13, .19] | .09 | -.11 | .69 |  | .01 [-.22, .24] | .12 | .01 | .93 |

Table S2 presents the results regarding the prediction of mothers’ reported parenting. There was no statistically significant prediction from the adolescents’ perceived autonomy support and controlling parenting, suggesting that mothers’ report would not change depending on how they were perceived by their adolescents. Interestingly, adolescents’ basic psychological needs satisfaction was significantly related with the change of mothers’ reported autonomy support. The higher basic psychological needs satisfaction of adolescents was at T1, the more their mother reported autonomy support. Combining the result from the main manuscript, there might be a positive cycle: The more mothers provide autonomy support to their adolescents, the higher their basic psychological needs satisfaction, which contributes to adolescents receiving more autonomy support from their mothers.

**Predictions of Depressive Symptoms and Basic Psychological Needs Satisfaction**

We conducted the same analyses in the main manuscript (Table 3), with the addition that we controlled for the effect of parent–child relationship satisfaction reported by both mothers and adolescents at T1 and age and sex of adolescents. The choice was based on the relations suggested by previous multi-informant studies (Korelitz & Garber, 2016; Taber, 2010; Tein et al., 1994) and their significant correlations with the variables we used in the main analyses. We presented the correlations between parent–child relationship satisfaction and the variables used in the main manuscript in Table S3.

| Table S3.  Correlations between Parent-child Relationship Satisfaction and the Variables Used in the Main Manuscript | | | |
| --- | --- | --- | --- |
|  | | Parent-child  Relationship Satisfaction (T1) | |
|  | | Reported by  mothers | Reported by adolescents |
| T1 | |  |  |
| Reported by mothers | |  |  |
|  | Autonomy support | .42* | .34* |
|  | Controlling parenting | -.29* | -.21* |
| Reported by adolescents | |  |  |
|  | Perceived autonomy support | .26* | .66* |
|  | Perceived control parenting | -.26* | -.44* |
|  | Basic psychological needs satisfaction | .22* | .38* |
|  | Depressive symptoms | -.23* | -.30* |
| Time 2 | |  |  |
| Reported by mothers | |  |  |
|  | Autonomy support | .37* | .38* |
|  | Controlling parenting | -.26* | -.18* |
| Reported by adolescents | |  |  |
|  | Perceived autonomy support | .24* | .55* |
|  | Perceived control parenting | -.27* | -.41* |
|  | Basic psychological needs satisfaction | .25* | .34* |
|  | Depressive symptoms | -.25* | -.33* |
| Control Variables | |  |  |
|  | Adolescents’ sex (Male = 0, Female = 1) | .04 | .20* |
|  | Adolescents’ age | .02 | .00 |
| *Note.* Correlation between mother-reported and adolescent-reported mother-child relationship Satisfaction was .41*. **p* < .05 | | | |

Although adolescents’ age was not significantly corelated with any variables (see Table 3 in the main manuscript), we included it as a control variable, given the influence of age on the accuracy of children’s reports in general (Taber, 2010).

As shown in Table S3, the results were basically the same as those reported in Table 3, except for the statistically significant relationship between controlling parenting reported by mothers and basic psychological needs satisfaction. It was not significant in the main manuscript (*B* = .07, β = .08, *p* = .07, shown in Table 3).

| Table S3.  *Predictions of Depressive Symptoms and Basic Psychological Needs Satisfaction* | | | | | | | | | |
| --- | --- | --- | --- | --- | --- | --- | --- | --- | --- |
| Predictor variable (T1) | (T2) | | | | | | | | |
|  | Basic psychological needs satisfaction | | | |  | Depressive symptoms | | | |
|  | *R*^2^ = .52 | | | |  | *R*^2^ = .47 | | | |
|  | *B* [95%CI] | *SE B* | β | *p* |  | *B* [95%CI] | *SE B* | β | *p* |
| Reported by mothers |  |  |  |  |  |  |  |  |  |
| Autonomy support | .12 [.02, 21] | .05 | .11 | .02 |  | -.03 [-.07, .02] | .02 | -.05 | .23 |
| Controlling parenting | .07 [.004, .14] | .04 | .09 | .04 |  | -.01 [-.05, .02] | .02 | -.03 | .49 |
| Reported by adolescents |  |  |  |  |  |  |  |  |  |
| Perceived autonomy support | -.00 [-.08, .07] | .04 | -.01 | .93 |  | .02 [-.02, .07] | .02 | .07 | .27 |
| Perceived control parenting | -.06 [-.12, .01] | .03 | -.08 | .10 |  | .04 [.00, .07] | .02 | .10 | .04 |
| Basic psychological needs satisfaction | .61 [.51, .72] | .05 | .59 | <.01 |  | -.08 [-.14, -.03] | .03 | -.15 | .01 |
| Depressive symptoms | -.21 [-.39, -.02] | .09 | -.10 | .03 |  | .54 [.44, .64] | .05 | .53 | <.01 |
| Control variables |  |  |  |  |  |  |  |  |  |
| Mother’s reported  parent-child relationship satisfaction | .05 [-.03, .14] | .04 | .06 | .23 |  | -.02 [-.07, .02] | .02 | -.04 | .36 |
| Adolescents’ reported parent-child relationship satisfaction | .01 [-.09, .11] | .05 | .01 | .84 |  | -.04 [-.09, .01] | .03 | -.09 | .12 |
| Adolescents’ sex | .01 [-.09, .11] | .05 | .01 | .83 |  | -.01 [-.06, .05] | .03 | -.01 | .82 |
| Adolescents’ age | .02 [-.04, .08] | .03 | .02 | .52 |  | .00 [-.03, .03] | .02 | .00 | .92 |

The mediational effects of adolescents’ basic psychological needs satisfaction on the relation between mothers’ reported autonomy support and adolescents’ depressive symptoms was statistically significant (-.01, 95%CI [-.02, -.001]), but not significant for mothers’ reported controlling parenting (-.01, 95%CI [-.01, .000]).

Although the relationship between controlling parenting reported by mothers and adolescents’ basic psychological needs satisfactions was weak and not robust, the result may suggest that parental behaviors reported by mothers and those perceived by adolescents had independent roles for predicting adolescents’ outcomes (Janssens et al., 2015; Nelemans et al., 2020). Outside the context of self-determination theory, some forms of parental control have been shown to be related with beneficial outcomes, including social adjustment and academic achievement (e.g., Ang, 2006; Leung et al., 1998; Paulson, 1994). In the present study, mother-reported controlling parenting itself may reflect a rather functional intention, such as monitoring and involvement (Barber et al., 1994), after controlling for adolescents’ perception.

## References

Ang, R. P. (2006). Effects of parenting style on personal and social variables for Asian adolescents. *American Journal of Orthopsychiatry, 76*(4), 503–511. <https://doi.org/10.1037/0002-9432.76.4.503>

Barber, B. K., Olsen, J. E., & Shagle, S. C. (1994). Associations between parental psychological and behavioral control and youth internalized and externalized behaviors. *Child Development, 65*(4), 1120–1136. <https://doi.org/10.2307/1131309>

Janssens, A., Goossens, L., Van den Noortgate, W., Colpin, H., Verschueren, K., & Van Leeuwen, K. (2015). Parents’ and adolescents’ perspectives on parenting: Evaluating conceptual structure, measurement invariance, and criterion validity. *Assessment, 22*(4), 473–489. <https://doi.org/10.1177/1073191114550477>

Korelitz, K. E., & Garber, J. (2016). Congruence of parents’ and children’s perceptions of parenting: A meta-analysis. *Journal of Youth and Adolescence, 45*(10), 1973–1995. <https://doi.org/10.1007/s10964-016-0524-0>

Leung, K., Lau, S., & Lam, W-L. (1998). Parenting styles and academic achievement: A cross-cultural study. *Merrill-Palmer Quarterly, 44*(2), 157–172. <https://www.jstor.org/stable/23093664>

Nelemans, S. A., Keijsers, L., Coplin, H., van Leerwen, K., Bijttebier, P., Verschueren, K., & Goossens, L. (2020). Transactional links between social anxiety symptoms and parenting across adolescence: Between- and within-person associations. *Child Development, 91*(3), 814–828. <https://doi.org/10.1111/cdev.13236>

Paulson, S. E. (1994). Relations of parenting style and parental involvement with ninth-grade students’ achievement. *Journal of Early Adolescence, 14*(2), 250–267. <https://doi.org/10.1177/027243169401400208>

Taber, S. M. (2010). The veridicality of children’s reports of parenting: A review of factors contributing to parent–child discrepancies. *Clinical Psychology Review, 30*(8), 999–1010. <https://dx.doi.org/10.1016/j.cpr.2010.06.014>

Tein, J -Y., Roosa, M. W., & Michaels, M. (1994). Agreement between parent and child reports on parental behaviors. *Journal of Marriage and the Family, 56*(2), 341–355. <https://doi.org/10.2307/353104>
